# A Systematic Review of Reporting Tools Applicable to Sexual and Reproductive Health Programmes: Step 1 in Developing Programme Reporting Standards

**DOI:** 10.1371/journal.pone.0138647

**Published:** 2015-09-29

**Authors:** Anna Kågesten, Ӧzge Tunçalp, Moazzam Ali, Venkatraman Chandra-Mouli, Nhan Tran, A. Metin Gülmezoglu

**Affiliations:** 1 Department of Population, Family and Reproductive Health, Johns Hopkins School of Public Health, Baltimore, Maryland, United States of America; 2 WHO Department of Reproductive Health and Research, including UNDP/UNFPA/UNICEF/WHO/World Bank Special Programme of Research, Development and Research Training in Human Reproduction, World Health Organization, Geneva, Switzerland; 3 Implementation Research Platform, Alliance for Health Policy and Systems Research, World Health Organization, Geneva, Switzerland; Florida International University, UNITED STATES

## Abstract

**Background:**

Complete and accurate reporting of programme preparation, implementation and evaluation processes in the field of sexual and reproductive health (SRH) is essential to understand the impact of SRH programmes, as well as to guide their replication and scale-up.

**Objectives:**

To provide an overview of existing reporting tools and identify core items used in programme reporting with a focus on programme preparation, implementation and evaluation processes.

**Methods:**

A systematic review was completed for the period 2000–2014. Reporting guidelines, checklists and tools, irrespective of study design, applicable for reporting on programmes targeting SRH outcomes, were included. Two independent reviewers screened the title and abstract of all records. Full texts were assessed in duplicate, followed by data extraction on the focus, content area, year of publication, validation and description of reporting items. Data was synthesized using an iterative thematic approach, where items related to programme preparation, implementation and evaluation in each tool were extracted and aggregated into a consolidated list.

**Results:**

Out of the 3,656 records screened for title and abstracts, full texts were retrieved for 182 articles, out of which 108 were excluded. Seventy-four full text articles corresponding to 45 reporting tools were retained for synthesis. The majority of tools were developed for reporting on intervention research (n = 15), randomized controlled trials (n = 8) and systematic reviews (n = 7). We identified a total of 50 reporting items, across three main domains and corresponding sub-domains: programme preparation (objective/focus, design, piloting); programme implementation (content, timing/duration/location, providers/staff, participants, delivery, implementation outcomes), and programme evaluation (process evaluation, implementation barriers/facilitators, outcome/impact evaluation).

**Conclusions:**

Over the past decade a wide range of tools have been developed to improve the reporting of health research. Development of Programme Reporting Standards (PRS) for SRH can fill a significant gap in existing reporting tools. This systematic review is the first step in the development of such standards. In the next steps, we will draft a preliminary version of the PRS based on the aggregate list of identified items, and finalize the tool using a consensus process among experts and user-testing.

## Introduction

Reporting of the key implementation elements of programmes in the field of sexual and reproductive health (SRH) is essential to understand the impact of the programmes, as well as to guide the efforts for future replication and scale-up. Indeed, readers of a programme report or publication need clear and complete information about the programme components, their development, implementation and evaluation, to be able to assess its quality as well as replicate the programme model [1]. However, the reality is that many programmes report on results and impacts without describing how, when, where and under what conditions programmes were developed and implemented [2]. In a systematic review on comprehensive adolescent health programmes inclusive of SRH services, Kågesten et al. [3] found substantial inconsistencies in the depth and scope of programme component descriptions. In both the peer-reviewed and grey literature, many publications and reports lacked a clear description of programme activities and their implementation. Consequently, programmes may demonstrate impact without providing details as to how results were obtained and how components can be replicated. The lack of an adequate description of implementation processes is not unique to program reporting, but widely recognized in relation to the reporting of clinical trials and other research designs. For example, Chalmers and Glasziou [4] estimated that over 30% of clinical trials and over 50% of planned study outcomes were not sufficiently described in publications, representing “billions of dollars” in avoidable reporting waste. Further analyses showed that between 40% and 89% of biomedical interventions were non-replicable because of inadequate description of intervention components [5].

The key underlying reason for varying quality and levels of details is the absence of standards for programme reporting in SRH. In 1996, the lack of adequate reporting on randomized clinical trials prompted the development of the Consolidated Standards of Reporting Trials (CONSORT) [6], and subsequent statements have been developed for reporting on study designs beyond randomized controlled trials such as non-randomized evaluations [7] and qualitative studies [8]. However, scholars increasingly emphasize the need for greater clarity on what and how to report in relation to programme preparation, implementation and evaluation processes to better facilitate replication and scale up irrespective of the study design used [2,9].

In response to this gap, the World Health Organization (WHO) Department of Reproductive Health and Research, including the UNDP/UNFPA/UNICEF/WHO/World Bank Special Programme of Research, Development and Research Training in Human Reproduction (HRP), in partnership with the Alliance for Health Policy and Systems Research hosted by the WHO, initiated a consultative process to develop Programme Reporting Standards (PRS) to be used by programme implementers and researchers in the field of SRH. The overall goal is to improve the quality of programme reporting in order to allow others to replicate the programme, as well as to better understand and document the success and barriers in its implementation. In line with recommendations for developing reporting guidelines provided by Moher et al [10], the current systematic review is the first step in the development of the PRS. The objectives of the systematic review are two-fold: 1) to provide an overview of available reporting guidelines and tools that have been used, or are suitable to use, for SRH programmes; and 2) identify core items used in programme reporting with a focus on programme preparation, implementation and evaluation processes, to be included in a draft tool.

### Defining key terms

Our primary interest for the present review is the reporting of programmes, whether by researchers or programmers. According to the Dictionary of Epidemiology [11], a programme is a “(formal) set of procedures to conduct an activity, e.g. control of malaria”, whereas an intervention study involves an “intentional change in some aspect of the status of subjects, e.g. introduction of a preventive or therapeutic regimen or an intervention designed to test a hypothesized relationship”. A programme may or may not be interventional in nature. However, because these terms are often taken to mean the same thing, we used the terms *programme* and *intervention* interchangeably to refer to a formal set of prevention, promotion and/or intervention activities. [1] We further used the term *programme components* in reference to the elements or activities that comprise a programme.

A key challenge in reviewing literature on reporting tools is the highly varying terminology used to describe programmes [1]. When describing individual studies, we therefore strived to retain the terminology used in the original publications. Finally, we used the terms *items* and *reporting items* interchangeably to refer to items included in reporting checklists or other tools (for example, the CONSORT statement has 21 items). Our goal was to identify a set of core items for potential inclusion in a PRS tool focused on SRH.

## Materials and Methods

We used a modified version of the Preferred Reporting Items for Systematic Reviews and Meta-Analyses (PRISMA) statement [12] to conceptualize and carry out the current systematic review. Each step of the review was specified in a protocol for the overall PRS project (the protocol was not published but it is available in S1 Text).

### Eligibility criteria

For the purpose of this review, we included any study or article that described a reporting guideline or tool that has been used, or would be suitable to use, for reporting on programmes in the field of SRH. In line with Moher et al [13], we defined a reporting guideline as a “checklist, flow diagram, or explicit text to guide authors in reporting a specific type of research”. Because the focus of our review extended beyond research reporting, we also included checklists or guiding texts developed for programme reporting outside of academia (e.g. by implementing organizations and donors). For the purposes of simplicity, from hereon we refer to all guidelines, checklists and other guiding specifications as “tools”. Finally, we included articles that outlined narrative recommendations for programme reporting, even if these did not present official tools. All included articles had to describe a tool or provide unique recommendations relevant to programme reporting, and be published between January 2000 and September 2014. We chose not to limit the search by programme or study design in order to capture as many relevant tools as possible. No language restrictions were applied.

As mentioned above, reporting tools had to have been used, *or be applicable to use*, for reporting on programmes targeting SRH outcomes. In line with the WHO’s mandate on SRH [14], such outcomes include but are not limited to: maternal mortality and morbidity, abortion, sexually transmitted infections and HIV prevention and treatment including mother-to-child transmission, adolescent pregnancy, family planning, safe abortion care, pregnancy and childbirth care, postnatal care of mother and newborn, and prevention and management of gender-based violence. By the term ‘applicable to use’, we mean tools used in the wider field of public health and medicine that may be relevant or suitable for SRH programmes even though the tools were not developed specifically for such outcomes (given that many of the issues central to programme reporting are not unique to the field of SRH). Two reviewers (AK, ÖT) evaluated which tools were applicable for inclusion. Those excluded were 1) tools that were minor modifications of an already established tool; 2) studies that merely assessed the quality of reporting or reviewed existing reporting tools; and 3) comments or editorials about a tool (unless these elaborated on items not otherwise included in existing tools).

### Information sources and search strategy

We searched six electronic databases: PubMed, Scopus, PsychInfo, Embase, MEDLINE and Global Health for the period January 2000 through September 2014. All database searches were run during the week of 1 September 2014. We developed a core search strategy combining MeSH terms with key words for use in PubMed. The strategy was built in three blocks: reporting tool/guideline AND programme/intervention AND SRH/Health, and further adapted according to the standards and relevant MeSH terms for each database. The full search strategy for each database is available in S1 Table.

For the grey literature, we conducted a focused search on identifying reporting tools used by donors. The selection of donors was based on those providing support to the HRP. Website searches of implementing organizations in the field of SRH were beyond the scope of the current review. Such organizations will, however, be included in the next steps of the PRS development. Other sources of data included reference lists of key articles and background documents [1,2,4,5,10,13,15], one example being a review about reporting guidelines in health research [13]. We also searched the library for the Enhancing the QUAlity and Transparency of Health Research (EQUATOR) network (http://www.equator-network.org/library/), a resource bank of reporting guidelines. The latter search was focused on tools for reporting on interventions and implementation.

### Study selection

Following the search process, two reviewers (AK, ÖT) divided all records and independently screened the titles and abstracts. Full texts were obtained for all articles that passed the initial screening. The same two reviewers (AK, ÖT) assessed all full texts, in duplicate, with inconsistencies resolved through discussion. Full texts were included if they met all inclusion criteria; all reasons for exclusion were recorded.

### Data extraction

Data was extracted using a standardized template across the following domains (see S2 Table for a detailed summary of the extracted data):

- Background details (author(s), year(s) of publication, journal(s) or other sources).
- Focus of tool (e.g. for reporting on a specific study design).
- Content area (e.g. for reporting in a specific field such as HIV).
- Number and description of reporting items included in the tool, or a summary of the recommended items for reporting if described in narrative format.
- Number and description of reporting items specific to programme preparation, implementation and evaluation.
- Validation (piloting or other modes of testing or validating the tool).

Each tool could have one or more sources; that is, data concerning the same tool could be extracted from different journal publications. One person in the research team (AK) extracted data from each included article. A second reviewer (ÖT) verified the extracted data for a random sample of 20% of included tools; inter-rater consistency (proportion of agreement) was over 95%.

### Synthesis of results

We applied an iterative, thematic approach to the synthesis of textual data from the included tools. Based on the number of items specific to programme preparation, implementation and evaluation, tools were initially ranked into high (all items or overall focus of tool), moderate (some items even if not the focus of the tool, or narrative discussion or relevant reporting themes) or low (one item or less, not the focus of the tool) relevance. In the first step, all extracted items were reviewed for their applicability to programme reporting and aggregated into a compiled list. This list included the original item, a brief description and its corresponding tool. In the next step, we used an inductive coding process where each item was coded according to its programme reporting domain (e.g. implementation outcome) and potential sub-domains (e.g. fidelity). We conducted iterative reviews of the extracted items to identify and refine domains and sub-domains, during which items and codes that were similar or redundant were merged. Items that were judged by the reviewers as inapplicable to programme preparation, implementation and evaluation processes were removed. This distinction was based on items already included in existing guidelines for reporting on study designs and results (e.g. CONSORT or non-randomized alternatives). The final list of items was organized according to their main corresponding domain and sub-domain. Because of the nature of the review, where the main focus was to provide a narrative description of items, the reporting of analytical comparative measures such as odds ratios were not applicable.

### Assessment of quality

Similar to previous systematic reviews of reporting guidelines [13], we did not appraise the methodological quality of tools, including of risk of bias within and across studies. The rationale for this was that the review sought to describe existing programme reporting tools rather than assess the quality of reporting, or the effectiveness or impact of programmes. As part of the synthesis process, there was an assessment on whether existing tools had been piloted or used widely based on the reported use in different geographical settings or endorsement by organizations and/or journals.

## Results

### Characteristics of included tools

**Fig 1:**
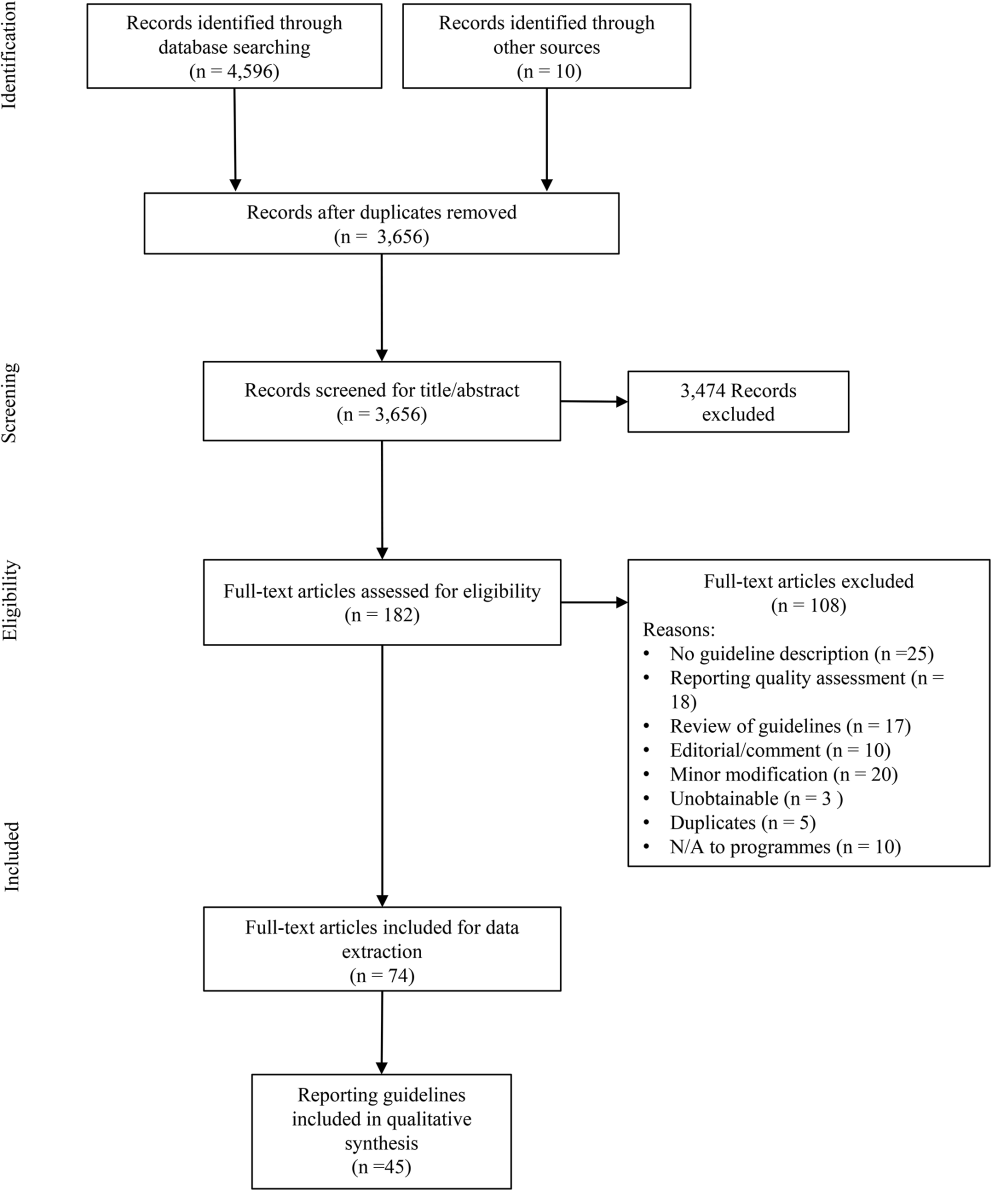
PRISMA 2009 Flowchart of screening and data extraction process. The majority of articles (96%) were published in peer-reviewed journals, the most common being BMC Medical Education or BMC Medical Research Methodology (8%), PLoS Medicine (7%) and Journal of Clinical Epidemiology (7%). The included articles corresponded to 45 tools (Table 1) retained for synthesis.

We screened the title and abstract of 3,656 records. Full texts were retrieved for 182 articles of which 108 were excluded; all reasons for exclusion were recorded. In total, 74 full text articles were retained for data extraction (Fig 1).

**Table 1:** Overview of included tools, by relevance to the current systematic review.

**High Relevance**				
**Tool (in alphabetical order)**	**Focus of tool**	**Content area**	**Nr of items**	**Source**
1. Complexity spectrum checklist	Randomized controlled trials	Complex intervention trials	14	[16]
2. CONSORT statement–proposed addition	Randomized controlled trials	Implementation reporting	N/A	[17]
3. CONSORT–SPI statement (on-going development)	Randomized controlled trials	Social and psychological intervention trials	N/A	[18,19]
4. CONSORT statement–unofficial extension	Randomized controlled trials	Behavioural medicine intervention trials	22	[20]
5. Reporting on development and evaluation of complex interventions in healthcare (CReDECI) guideline	Intervention research, all study designs	Development and evaluation of complex interventions in healthcare	16	[21,22]
6. Guidelines for reporting evidence-based practice educational interventions (GREET) statement (on-going development)	Intervention research, all study designs	Description of educational evidence-based practice strategies	N/A	[23–25]
7. Implementation research framework for health sciences	Implementation research	Framework for implementation research in health	N/A	[9]
8. Oxford Implementation Index	Systematic reviews	Implementation data in systematic reviews	17	[26]
9. Program evaluation and monitoring system (PEMS)	Program evaluation and monitoring	HIV Prevention	8	[27]
10. PROGRESS-Plus checklist	Intervention research, all study designs	Equity lens for reporting on interventions	N/A	[28,29]
11. Reporting of HIV interventions	Intervention research, all study designs	Quality of study methods in HIV prevention interventions	11	[30]
12. Reporting of implementation for injury prevention initiatives	Systematic reviews	Injury prevention implementation	N/A	[31]
13. Reporting of nursing interventions	Intervention research, all study designs	Content of complex nursing interventions	20	[32]
14. Reporting of public health interventions	Intervention research, all study designs	Public health interventions	N/A	[33]
15. Reporting of tailored interventions	Intervention research, all study designs	Tailored interventions	7	[34]
16. Structured assessment of feasibility (SAFE) checklist	Intervention research, all study designs	Feasibility of complex interventions in mental health services	16	[35,36]
17. Standards for quality improvement reporting excellence (SQUIRE) guidelines	Intervention research, all study designs	Quality improvement interventions	19	[37–39]
18. Integrated checklist for determinants of practice (TICD)	Determinants of practice	Health care and chronic disease	53	[40]
19. Template for intervention description and replication (TIDieR) statement	Intervention research, all study designs	Description of interventions	12	[41]
20. Transparent Reporting of Evaluations with Nonrandomized Designs (TREND) statement	Intervention research, non-randomized design	Evaluation of public health/behavioural interventions	22	[7]
21. Workgroup for intervention development and evaluation research (WIDER) statement	Intervention research, all study designs	Components of behaviour change interventions	24	[42,43]
**Moderate Relevance**				
**Tool (in alphabetical order)**	**Focus of tool**	**Content area**	**Nr of items**	**Source**
22. CONSORT statement	Randomized controlled trials	General	25	[6,44–49]
23. CONSORT statement–extension	Randomized controlled trials	Non-pharmacological treatments	22	[50]
24. CONSORT–EHEALTH statement	Intervention research, all study designs	Evaluations of web-based and mobile health interventions	25	[51,52]
25. CONSORT statement–extension	Randomized controlled trials	Pragmatic trials	22	[53]
26. Consolidated criteria for reporting qualitative research (COREQ) statement	Qualitative studies	Interviews and focus groups	32	[8]
27. Guidance for Reporting Involvement of Patients and Public (GRIPP) checklist	Patients and public involvement in research	Health technology/health services	10	[54]
28. Preferred reporting standards in systematic reviews and meta-analyses (PRISMA) statement	Systematic reviews	General	27	[12,55]
29. PRISMA–Equity statement	Systematic reviews	Health equity	27	[56–59]
30. Reporting of internet interventions	Intervention research, all study designs	Internet	12	[60]
31. Reporting of public health programs in Colorado	Program reporting	Public health program reporting system	N/A	[61]
32. Statement on reporting of evaluation studies in health informatics (STARE–HI)	Evaluation studies	Evaluation of health informatics systems	14	[62,63]
**Low Relevance**				
**Tool (in alphabetical order)**	**Focus of tool**	**Content area**	**Nr of items**	**Source**
33. Checklist for systematic reviews of non-randomized studies	Systematic reviews, non-randomized designs	Non-randomized studies of health care interventions	4	[64]
34. Consolidated Health Economic Evaluation Reporting Standards (CHEERS) statement	Intervention research, economic evaluations	Economic evaluations of health care interventions	24	[65,66]
35. Checklist to evaluate report of non-pharmacological trials (CLEAR NPT)	Randomized controlled trials	Non-pharmacological treatments	10	[67]
36. Enhancing transparency in reporting the synthesis of qualitative research (ENTREQ)	Systematic reviews	Qualitative research synthesis	21	[68]
37. International Society for Pharmacoeconomics and Outcomes Research checklist for cost-effectiveness analysis alongside clinical trials (ISPOR RCT-CEA)	Randomized controlled trials	Cost-effectiveness alongside clinical trials	27	[69]
38. Reporting guidelines for observational longitudinal studies	Observational studies	Longitudinal health and medical research	33	[70]
39. Reporting guidelines for survey research	Survey research	General	38	[71]
40. Reporting qualitative research in health informatics (REQ-HI) recommendations	Qualitative research	Health informatics	14	[72]
41. Rural and Remote Health Journal guideline	All study designs	Rural and remote health	15	[73]
42. Standards for reporting on diagnostic accuracy studies (STARD) statement	Diagnostic accuracy studies	General	25	[74–77]
43. Strengthening reporting of genetic associations (STREGA) statement	Observational	Genetic association studies	22	[78]
44. STandards for Reporting Interventions in Clinical Trials of Acupuncture (STRICTA) statement	Intervention research, all study designs	Acupuncture	6	[79,80]
45. STrengthening the Reporting of OBservational studies in Epidemiology (STROBE statement)	Observational	General	22	[81,82]

Note: N/A means that the tool did not present an official list of items, but included a narrative description of important reporting elements.

The majority of tools were developed for reporting on intervention research (n = 15), randomized controlled trials (n = 8) and systematic reviews (n = 7). Other guideline focuses included observational studies (n = 3), diagnostic accuracy (n = 2), qualitative studies (n = 2), survey research (n = 1), general study designs (n = 1), determinants of practice (n = 1), patient/public involvement in research (n = 1), programme evaluation and monitoring (n = 1) programme reporting (n = 1), evaluation studies (n = 1) and implementation research (n = 1).

Reporting tools covered a wide range of content areas such as behaviour change, health informatics, mobile or e-health, equity, nursing and complex interventions. Of the included tools, three described reporting items specific to SRH (for example, details on sexual partners or HIV status) [27–30]. The majority of tools presented a checklist of core items for reporting (ranging from 6–58 items), while others (n = 8) used a narrative description of essential reporting elements. About half the tools did not include items or topics specific to the description of programme preparation, implementation or evaluation. However, some tools listed items or included a narrative description of topics that could be indirectly related to these topics, such as a description of unexpected events that in turn may affect implementation.

Overall, 21 tools were ranked as having a high relevance to the present review, 11 were ranked as moderate and 13 as low relevance in line with the criteria described earlier. Four tools are especially worth mentioning because of their high relevance and recent publication. First, the template for intervention description and replication (TIDieR) [41] published in 2014 provide an itemized checklist for reporting on intervention studies, including items such as the intervention name, rationale, materials, procedures (how, by whom, when and where delivery occurred), as well as the dose, modifications and fidelity to the intervention. Secondly, in 2013 the workgroup for intervention development and evaluation research (WIDER) [42] outlined a number of recommendations for describing the development, content, setting, mode of delivery, intensity, duration, and fidelity of behavior change interventions. Thirdly, in 2013 Peters et al [9] proposed a set of guiding questions for reporting implementation research, including the description of implementation strategies, context, complexity and real-world conditions. The implementation terminology presented as part of this framework [9] was also used to organize the findings from the current review. Finally, in 2003, Davidson et al [20] provided eight recommendations for minimal detail in the reporting of behavioral medicine interventions, including content/elements, provider, format, setting, recipient, intensity, duration and fidelity.

### Description of items

A total of 226 items related to programme preparation, implementation and evaluation processes were extracted and further consolidated into 50 items for potential inclusion in a PRS tool for SRH programmes. Items that were similar across multiple tools were merged, and the wording of items was changed accordingly. Where applicable, the wording was also changed from *intervention* to *programme* to better correspond to the purposes of the current review. The final list of items, their descriptions, corresponding domains and sub-domains, and sources are presented in Table 2.

**Table 2:** Reporting items related to programme preparation, implementation and evaluation.

Domain	Sub-domain	Item	Description
Programme preparation	Objective/Focus	1. Programme name	Name of programme [41].
		2. Objectives and anticipated impact of programme (why)	Anticipated short-term and long-term influences of programme on individual participants as well as wider implications [16,30].
		3. Target population	Characteristics of the target population planned to be reached and at what level (individual, group, wider population) [9,20,26–29,32,35,36,71].
	Design	4. Organization/agency	Mention the name, credentials and affiliations of the organization(s) developing the programme [9,33,51,52].
		5. Funding source	Name of programme donor/funding source(s) [37,38,44,51,52,65,66,81,82].
		6. Programme design process	Description of the process of designing the programme [37,38,42].
		7. Theoretical foundation	Underlying theory and/or logic model of the programme [7,32,33,41–43], with details for how this theory guided the programme design and messages [34].
		8. Program manual	Whether a manual or protocol existed for the programme [35,36], and where this can be accessed [62,63].
		9. Implementation strategy	Details on whether an implementation strategy was developed [9,21,22,30], and if any research questions were specific to implementation [9].
		10. Evaluation plans	Detail any evaluation plans, both to assess programme implementation/process and to evaluate the programme’s impact/results [33,38,41].
	Piloting	11. Piloting of activities	Whether programme activities were piloted, and if so detail how, when, by whom and the results [21,22,33,35,36].
Programme implementation	Content	12. Components/activities	Define and describe the content of programme activities in enough detail to allow replication [7,9,12,16,18,20,22,25–27,30,32,35,36,41,42,44,45,53,60,61,69,71]. If a control group was used, the content of any activities assigned to the control should also be described [12,18,20,42,53,67].
		13. Complexity	Degree of complexity of the activities, such as whether single or multiple components were included [16].
		14. Standardisation	Whether the content of components/activities followed a standardised protocol or curriculum [67].
		15. Innovation	Degree of innovation as part of the programme [21,30].
		16. Materials	Type of materials used [24,25,41] and where these can be accessed if applicable [32,41,42].
	Timing, duration, location	17. Timing (when)	Timing and duration of the programme (start and finish) [7,16,18,20,41,60,71].
		18. Setting (where)	Key aspects of the programme setting [7,9,16,20,26,27,31,32,41,42,53,62,63,83], including geographical context (e.g. country, rural/urban) [73], single/multiple locations [16], type of context [27,41,54] such as “real-world” or clinical [41], and any infrastructure required [9,41].
		19. Dose and intensity (how much)	Number of sessions/activities, how often activities were delivered [18,26,27,32,41,60], whether the frequency of activities was predetermined or varying [18], and the intensity or duration of each activity [7,18,20,41,42].
	Providers/staff	20. Provider characteristics (Who)	Organization(s)/agencies involved in delivering the programme activities [26,27] (name and type) [27], number of staff and their responsibilities [16], staff characteristics including demographics, professions, experience, education and technical skills required [7,8,16,18,20,26,30,32,41,42,67,74,75].
		21. Provider/staff training	Details on how programme staff was recruited, trained and supervised to deliver activities (when, how and by whom) [18,25,26,35,36].
		22. Provider reflexivity	Reflection about the relationship between providers and participants, such as whether participants knew the staff [8], influences of professional opinions and the self-efficacy of providers[40].
	Participants	23. Participant recruitment	Process of recruiting programme participants [18,71].
		24. Participants (to whom)	Characteristics of participants that actually received the programme [20,32,42,54,61,69]. Report subgroups by key demographic factors such as age, biological sex/gender, socioeconomic status, education level, religion [9,20,26–29,32,35,36,56,59], HIV status, and nr of sexual partners [27]. Note participant risk profiles, if any (e.g. disadvantaged populations) [27,56,59].
		25. Participant preparation	Whether anything was done to prepare or brief participants prior to the start of the programme [25,32].
	Delivery	26. Methods used to deliver activities (how)	Specific methods/channels used for delivering programme activities [7,18,20,26,27,41,42,60,74,75], degrees of human interactivity [16,25,60], level of involvement [54], technology required [26].
		27. Efforts to ensure fidelity of participants	Efforts to ensure fidelity, increase participation, compliance or adherence, and reduce contamination [7,18,24,25,32,40,41], such as incentives or compensation [7,17,18,24,25,32,40,41].
		28. Efforts to ensure fidelity of providers/staff	Efforts to enhance adherence of providers [18,26,35,36] such as staff meetings [26], support [26,35], incentives [18,40], feedback [18], motivation [26] and supervision [35].
	Implementation outcomes	29. Acceptability	Perception and comfort among stakeholders about the programme, its relative advantage and credibility [9].
		30. Appropriateness	Perceived fit or relevance of the intervention as judged by the implementers [9].
		31. Feasibility/practicality	The actual fit, utility or suitability of the programme for the everyday life of participants [9,18].
		32. Adoption	Uptake/utilization of programme [9,18,26]. Difference in uptake by intervention or control groups, if applicable [18].
		33. Coverage/Reach	The spread or penetration of the programme components [9,60].
		34. Attrition	Non-participation and dropout of participants [18], along with reasons for why [8,18].
		35. Unexpected end of programme	Whether the programme ended or stopped earlier than planned, along with reasons for why [45].
		36. Reversibility	Whether it would be possible to stop the programme without negative or harmful effects [35,36].
		37. Contamination of activities	Unanticipated spread of activities outside of the programme target population [18,26].
		38. Fidelity	Whether the programme was delivered as intended, e.g. discrepancies between the programme design and the actual implementation of components and methods in the "real life context" [7,9,17–22,31–33,38,41,42].
		39. Reasons for low fidelity	Reasons for any deviation from planned activities or others parts of the programme design [41].
		40. Sustainability	Extent to which participants may be able to use the programme in their everyday life, for example whether any support structures are in place to maintain behaviour changes [18,25], what happened after the program [61], whether any follow-up sessions are planned [25].
		41. Costs of implementation	Costs and required resources for implementation [9,21,35,36,69], including time, human resources, materials, set-up, administration [35], delivery strategy [9].
Programme evaluation	Process evaluation	42. Process or implementation evaluation methods	Method that was used to assess implementation outcomes [21,22]. For example, how fidelity was monitored and measured [20].
		43. Effect of implementation process on results	Whether the implementation process affected results and quality of the programme results [21,22].
		44. External events affecting implementation	Significant external events occurring at the time of intervention (e.g. social political, economic and/or geographical), which might have affected the implementation [9,17,19,21,33,38,54,62,63].
		45. Ethical considerations	Ethical issues that might have affected the implementation [26].
	Implementation barriers and facilitators	46. Implementation barriers and facilitators	Detailed description of factors hindering and facilitating implementation of the programme [7,17,21,31,38].
		47. Strengths and limitations	Appraise weaknesses [33] and strengths [31] in the programme design, what worked and what can be improved [33].
	Impact/results evaluation	48. Outcome evaluation methods	How programme results/impact was evaluated [21,22,35,36,38], differentiating between effectiveness, efficacy and cost savings [35].
		49. Unexpected/negative effects	Any unexpected and/or negative effects of the programme [33,41].
		50. Differential effects	Whether the programme effects differed according based on characteristics such as biological sex/gender, ethnicity, socioeconomic status, age, geographic location [33,35].

The items were organized according to three main domains: 1) programme preparation, 2) program implementation, and 3) programme evaluation processes. A number of corresponding sub-domains were also identified. The following section provides a brief description of each domain and sub-domain.

#### Programme preparation

Three sub-domains were identified which related to programme preparation or planning. These include the programme’s *objective/focus* (overall goal, anticipated impact, and target population); how the programme was *designed* (organization(s) and donors involved in developing the program, theory of change or logic model, the process of designing programme activities, existence of a manual/protocol and implementation strategy); and *piloting* (whether and how activities were piloted, along with results from the pilot).

#### Programme implementation

Six sub-domains emerged related to programme implementation. *Programme content* refers to the actual content of programme activities described with enough detail to allow replication; the complexity, number, level and innovation of activities; materials used and where to locate these. *Timing*, *duration and location* include items describing when and where programme activities were delivered, and the dose and intensity of activities. *Programme providers/staff* refer to who conducted the activities, as well as the training, characteristics, responsibilities and reflexivity of delivering staff. *Programme participants* cover who the actual recipients were; how participants were recruited and any preparation prior to the start of activities. Furthermore, *programme delivery* items describe how the programme activities were delivered; materials used; and efforts to ensure fidelity of both participants and staff. Finally, *programme implementation outcomes* refer to the actual acceptability and adoption of the programme; its coverage/reach, feasibility, modification, fidelity and reasons for low fidelity; appropriateness, implementation costs, reversibility, sustainability and unexpected events among other items.

#### Programme evaluation

With regard to programme evaluation processes, three sub-domains were identified. *Process or implementation evaluation* includes items describing process evaluation methods; how the implementation process might have affected results; contextual/external events; and ethical considerations affecting implementation. *Implementation barriers and facilitators* relate to factors hindering or facilitating implementation, as well as appraised strengths and limitations of the overall programme. Finally, *impact/outcome evaluation* items describe the process of evaluating programme outcomes (differentiating between effectiveness, efficacy and cost savings) or any upcoming evaluation plans, and whether the programme had any unexpected negative or differential effects.

## Discussion

While there is growing evidence about “what works” to improve SRH outcomes, less is known about “how-to” implement, replicate and scale-up programmes [9]. Many programmes describe outcomes and results but do not provide enough detail to allow others to understand what exactly was done, the evidence, and lessons learnt from implementation barriers and/or facilitators. The current systematic review sought to provide an overview of tools that may be used for reporting on SRH programmes, and to further identify core items for programme reporting.

We found that over the past decade a wide range of tools have been developed to improve the reporting of health research. Most of the identified tools were essentially guidelines for reporting on research study design and results and included none or few items relevant to programme reporting. A number of tools were, however, of greater relevance. In particular, recent tools such as TIDieR [41] and WIDER [42] may substantially improve the reporting of interventions using both randomized and non-randomized designs. For example, the TIDieR checklist [41] is a comprehensive list of items for reporting on how, where, when, by whom and with what fidelity interventions were implemented, thus moving beyond the single item provision of “sufficient” details on the intervention in order to allow replication used in guidelines such as CONSORT.

Nevertheless, these tools were developed specifically for intervention *research* and reporting in peer-reviewed journals. Although there seem to be increasing numbers of programme evaluations published in peer-reviewed journals and the existence of journals for this purpose (e.g. *Global Health*: *Science and Practice*), it is probably fair to assume that a significant proportion of programme reports are published on the web or in print outside the peer-reviewed journals. Deficiencies remain in the above frameworks for programme reporting, specifically as it relates to reporting of implementation strategies and outcomes. As recently noted by Glasziou et al [5], there is need for improved reporting beyond peer-reviewed journals that focus “more broadly at the multiple and various forms in which research processes and findings are reported”. Many programmes operate under complex, real world conditions making it difficult to communicate exactly what is being done, and how, in a timely and consistent manner. Coordinators, implementers, managers as well as researchers thus need a standardized way of documenting and reporting implementation strategies and outcomes throughout the course of the programme so that others can learn from their experiences. As a result, there is need for guidance for complete and accurate reporting on programme preparation, implementation and evaluation processes in real world contexts.

The current systematic review is the first step in the development of PRS for SRH, where we sought to provide a consolidated list of the types of items included in existing tools. While there was substantial diversity in the focus, scope and relevance of the tools reviewed, we identified 50 items related to the description of programme preparation, implementation and evaluation processes. Additional items and themes may be identified and suggested during subsequent steps of the PRS development. Specifically, in line with the recommendations by Moher et al [10], we will conduct a Delphi consensus exercise with a panel of experts to review and add to the list of items. This will be followed by a face to face consultative meeting to further refine and discuss the items, finalize the PRS and plan for its implementation. Finally, the PRS will be pilot-tested for user feasibility via different SRH programmes supported by the WHO. The specific purpose of the final PRS will be to help programme staff and researchers write reports and communicate key elements about how the programme was prepared, implemented and evaluated. The intended users of the PRS include programme staff and implementers writing reports to donors or for dissemination to external audiences. The PRS may also serve as a guide on what to include in peer-reviewed publications about programmes and their implementation processes. Finally, the PRS tool may function as a guide for upfront programme planning and implementation by outlining the essential elements that need to be reported on.

While every effort was made to undertake a comprehensive, systematic search of relevant literature, there are some limitations to the review. Because the review was restricted to peer-reviewed literature and selected grey literature sources published in the last 15 years it is possible that we missed relevant programme reporting tools that were published before or after this timeframe, or that may not be available to the public. One example is the Standards for Reporting Implementation Studies of Complex Interventions (StaRI) [84], which was recently published (2015) and therefore not captured by the current systematic search. We attempted to minimize this risk of bias by piloting our search strategy and implemented this in a number of databases relevant to SRH. We also searched the EQUATOR database, which provides a comprehensive listing of available reporting tools. Despite the use of a structured, piloted data extraction form it is possible that we overlooked some items. Finally, frameworks on developing a scale-up strategy, such as the WHO ExpandNet tool [85] and the SURE checklist for identifying factors affecting the implementation of a policy option [86], were beyond the scope of the current review based on its inclusion and exclusion criteria. We acknowledge the importance and utility of these tools and frameworks for programmes and therefore they may inform the subsequent steps of the PRS development.

As far as we know, this is the first systematic review of tools and items relevant to reporting of SRH programmes. The review thus fills an important gap in the literature on programme reporting.

## Conclusions

Over the last decade a number of tools for reporting of research have been published. Recent initiatives have focused on improving the reporting of intervention research through guidelines such as TiDiER [13] and WIDER [12]. However, few tools include specific elements related to the description of programme preparation and implementation, and we could not locate any standardized tools for reporting of programmes in the field of SRH. Development of PRS for SRH programmes can therefore fill a significant gap in existing reporting tools. Specifically, the availability of PRS can help improve descriptions of programme preparation, implementation and evaluation processes, which in turn can guide replication and scale-up of successful models. This systematic review is the first step in the development of such standards; in the next steps, we will draft a preliminary version of the PRS based on the aggregate list of identified items, and finalize the tool using a consensus process among experts and user-testing.

## Supporting Information

S1 Table Search strategies. (DOCX)

S2 Table Data extraction overview. (XLSX)

S1 Text Systematic Review Protocol. (PDF)
