# Supplementary material for: A Systematic Review of Reporting Tools Applicable to Sexual and Reproductive Health Programmes: Step 1 in Developing Programme Reporting Standards
Source: PLoS One. 2015 Sep 29;10(9):e0138647. doi: 10.1371/journal.pone.0138647 (PMC4852887; doi:10.1371/journal.pone.0138647)
Supplement: S1 Table — (DOCX) [file pone.0138647.s001.docx]

**S1 Table. Database search strategies**

**Core search strategy**

| 1. **Reporting tool/guideline** | |
| --- | --- |
| Key words: | Reporting guideline*[tw] OR reporting tool*[tw] OR reporting guidance[tw] OR reporting standard*[tw] OR reporting framework*[tw] Reporting of implementation*[tw] OR reporting of intervention*[tw]  OR reporting checklist*[tw] OR completeness or reporting[tw] |
| Controlled vocabulary: | Checklist*[Mesh] OR Publishing/standards*[Mesh] OR |
| **AND** | |
| 1. **Programme/intervention** | |
| Key words: | Programme[tw] OR program[tw] OR programmes[tw] OR programs[tw] OR intervention[tw] OR interventions[tw] OR implementation[tw] OR implementation research[tw] OR study[tw] OR studies[tw] |
| Controlled vocabulary: | Program development*[Mesh] |
| **AND** | |
| 1. **Applicable to SRH/health** | |
| Key words: | Reproductive health*[tw] OR sexual health*[tw] OR sexuality[tw] OR sexual[tw] OR reproductive[tw] OR maternal[tw] OR maternal health*[tw] OR public health[tw] OR health[tw] OR medical[tw] OR epidemiology[tw] |
| Controlled vocabulary: | Reproductive Health*[Mesh] |

**Full strategies (database specific)**

All searches were conducted and imported on September 2, 2014

| **PubMed** |
| --- |
| (((((((Reporting guideline*[tw] OR reporting tool*[tw] OR reporting guidance[tw] OR reporting standard*[tw] OR reporting framework*[tw] Reporting of implementation*[tw] OR reporting of intervention*[tw] OR reporting checklist*[tw] OR Checklist*[Mesh] OR Publishing/standards*[Mesh]) AND (Programme[tw] OR program[tw] OR programmes[tw] OR programs[tw] OR intervention[tw] OR interventions[tw] OR implementation[tw] OR implementation research[tw] OR study[tw] OR studies[tw] OR (Program development*[Mesh]) AND (Reproductive health*[tw] OR sexual health*[tw] OR sexuality[tw] OR sexual[tw] OR reproductive[tw] OR maternal[tw] OR maternal health*[tw] OR public health[tw] OR health[tw] OR medical[tw] OR epidemiology[tw] OR Reproductive Health*[Mesh])))))) Filters: Publication date from 2000/01/01 to 2014/12/31 |

| **Psychinfo** |
| --- |
| http://search.ebscohost.com.ezproxy.welch.jhmi.edu/login.aspx?direct=true&db=psyh&bquery=(Reporting+guideline*+OR+reporting+tool*+OR+%26quot%3breporting+guidance%26quot%3b+OR+reporting+standard*+OR+reporting+framework*+OR+%26quot%3breporting+of+implementation%26quot%3b+OR+%26quot%3breporting+of+intervention%26quot%3b+OR+reporting+checklist*+OR+%26quot%3bcompleteness+of+reporting%26quot%3b)+AND+(((DE%26quot%3b+program+development%26quot%3b))+OR+(Programme+OR+program+OR+programs+OR+programmes+OR+intervention+OR+interventions+OR+implementation+OR+%26quot%3bimplementation+research%26quot%3b+OR+study+OR+studies))+AND+(((DE+%26quot%3bReproductive+health%26quot%3b)+OR+(DE+%26quot%3bsexuality%26quot%3b)+OR+(DE+%26quot%3bpublic+health%26quot%3b))+OR+(Reproductive+health*+OR+sexual+health*+OR+maternal+health*+OR+maternal+OR+sexual+OR+sexuality+OR+reproductive+OR+%26quot%3bpublic+health%26quot%3b+OR+health+OR+medical+OR+epidemiology))&cli0=PY&clv0=200001-201412&type=1&site=ehost-live&scope=site |

| **Embase/MEDLINE** |
| --- |
| ((TITLE-ABS-KEY("reporting guideline") OR TITLE-ABS-KEY("reporting tool") OR TITLE-ABS-KEY("reporting guidance") OR TITLE-ABS-KEY ("reporting standard") OR TITLE-ABS-KEY("reporting framework") OR TITLE-ABS-KEY("reporting of implementation") OR TITLE-ABS-KEY("reporting of intervention") OR TITLE-ABS-KEY("reporting checklist") OR TITLE-ABS-KEY("completeness of reporting" ))) AND ((TITLE-ABS-KEY(programme*) OR TITLE-ABS-KEY(program*) OR TITLE-ABS-KEY(programs) OR TITLE-ABS-KEY(programmes) OR TITLE-ABS-KEY(intervention) OR TITLE-ABS-KEY(interventions) OR TITLE-ABS-KEY(implementation) OR TITLE-ABS-KEY("implementation research") OR TITLE-ABS-KEY(study) OR TITLE-ABS-KEY(studies))) AND ((TITLE-ABS-KEY("reproductive health") OR TITLE-ABS-KEY("sexual health") OR TITLE-ABS-KEY("maternal health") OR TITLE-ABS-KEY(maternal) OR TITLE-ABS-KEY(sexual) OR TITLE-ABS-KEY(sexuality) OR TITLE-ABS-KEY(reproductive) OR TITLE-ABS-KEY("public health") OR TITLE-ABS-KEY(health) OR TITLE-ABS-KEY(medical) OR TITLE-ABS-KEY(epidemiology))) AND ( LIMIT-TO(PUBYEAR,2014) OR LIMIT-TO(PUBYEAR,2013) OR LIMIT-TO(PUBYEAR,2012) OR LIMIT-TO(PUBYEAR,2011) OR LIMIT-TO(PUBYEAR,2010) OR LIMIT-TO(PUBYEAR,2009) OR LIMIT-TO(PUBYEAR,2008) OR LIMIT-TO(PUBYEAR,2007) OR LIMIT-TO(PUBYEAR,2006) OR LIMIT-TO(PUBYEAR,2005) OR LIMIT-TO(PUBYEAR,2014) OR LIMIT-TO(PUBYEAR,2013) OR LIMIT-TO(PUBYEAR,2012) OR LIMIT-TO(PUBYEAR,2011) OR LIMIT-TO(PUBYEAR,2010) OR LIMIT-TO(PUBYEAR,2009) OR LIMIT-TO(PUBYEAR,2008) OR LIMIT-TO(PUBYEAR,2007) OR LIMIT-TO(PUBYEAR,2006) OR LIMIT-TO(PUBYEAR,2005) OR LIMIT-TO(PUBYEAR,2004) OR LIMIT-TO(PUBYEAR,2003) OR LIMIT-TO(PUBYEAR,2002) OR LIMIT-TO(PUBYEAR,2001) OR LIMIT-TO(PUBYEAR,2000) OR LIMIT-TO(PUBYEAR,2014) OR LIMIT-TO(PUBYEAR,2013) OR LIMIT-TO(PUBYEAR,2012) OR LIMIT-TO(PUBYEAR,2011) OR LIMIT-TO(PUBYEAR,2010) OR LIMIT-TO(PUBYEAR,2009) OR LIMIT-TO(PUBYEAR,2008) OR LIMIT-TO(PUBYEAR,2007) OR LIMIT-TO(PUBYEAR,2006) OR LIMIT-TO(PUBYEAR,2005) OR LIMIT-TO(PUBYEAR,2004) OR LIMIT-TO(PUBYEAR,2003) OR LIMIT-TO(PUBYEAR,2002) OR LIMIT-TO(PUBYEAR,2001) OR LIMIT-TO(PUBYEAR,2000) ) |

| **Scopus** |
| --- |
| ((TITLE-ABS-KEY("reporting guideline") OR TITLE-ABS-KEY("reporting tool") OR TITLE-ABS-KEY("reporting guidance") OR TITLE-ABS-KEY ("reporting standard") OR TITLE-ABS-KEY("reporting framework") OR TITLE-ABS-KEY("reporting of implementation") OR TITLE-ABS-KEY("reporting of intervention") OR TITLE-ABS-KEY("reporting checklist") OR TITLE-ABS-KEY("completeness of reporting" ))) AND ((TITLE-ABS-KEY(programme*) OR TITLE-ABS-KEY(program*) OR TITLE-ABS-KEY(programs) OR TITLE-ABS-KEY(programmes) OR TITLE-ABS-KEY(intervention) OR TITLE-ABS-KEY(interventions) OR TITLE-ABS-KEY(implementation) OR TITLE-ABS-KEY("implementation research") OR TITLE-ABS-KEY(study) OR TITLE-ABS-KEY(studies))) AND ((TITLE-ABS-KEY("reproductive health") OR TITLE-ABS-KEY("sexual health") OR TITLE-ABS-KEY("maternal health") OR TITLE-ABS-KEY(maternal) OR TITLE-ABS-KEY(sexual) OR TITLE-ABS-KEY(sexuality) OR TITLE-ABS-KEY(reproductive) OR TITLE-ABS-KEY("public health") OR TITLE-ABS-KEY(health) OR TITLE-ABS-KEY(medical) OR TITLE-ABS-KEY(epidemiology))) AND ( LIMIT-TO(PUBYEAR,2014) OR LIMIT-TO(PUBYEAR,2013) OR LIMIT-TO(PUBYEAR,2012) OR LIMIT-TO(PUBYEAR,2011) OR LIMIT-TO(PUBYEAR,2010) OR LIMIT-TO(PUBYEAR,2009) OR LIMIT-TO(PUBYEAR,2008) OR LIMIT-TO(PUBYEAR,2007) OR LIMIT-TO(PUBYEAR,2006) OR LIMIT-TO(PUBYEAR,2005) OR LIMIT-TO(PUBYEAR,2014) OR LIMIT-TO(PUBYEAR,2013) OR LIMIT-TO(PUBYEAR,2012) OR LIMIT-TO(PUBYEAR,2011) OR LIMIT-TO(PUBYEAR,2010) OR LIMIT-TO(PUBYEAR,2009) OR LIMIT-TO(PUBYEAR,2008) OR LIMIT-TO(PUBYEAR,2007) OR LIMIT-TO(PUBYEAR,2006) OR LIMIT-TO(PUBYEAR,2005) OR LIMIT-TO(PUBYEAR,2004) OR LIMIT-TO(PUBYEAR,2003) OR LIMIT-TO(PUBYEAR,2002) OR LIMIT-TO(PUBYEAR,2001) OR LIMIT-TO(PUBYEAR,2000) OR LIMIT-TO(PUBYEAR,2014) OR LIMIT-TO(PUBYEAR,2013) OR LIMIT-TO(PUBYEAR,2012) OR LIMIT-TO(PUBYEAR,2011) OR LIMIT-TO(PUBYEAR,2010) OR LIMIT-TO(PUBYEAR,2009) OR LIMIT-TO(PUBYEAR,2008) OR LIMIT-TO(PUBYEAR,2007) OR LIMIT-TO(PUBYEAR,2006) OR LIMIT-TO(PUBYEAR,2005) OR LIMIT-TO(PUBYEAR,2004) OR LIMIT-TO(PUBYEAR,2003) OR LIMIT-TO(PUBYEAR,2002) OR LIMIT-TO(PUBYEAR,2001) OR LIMIT-TO(PUBYEAR,2000) ) |

| **Global Health** |
| --- |
| ((reporting guideline* or reporting tool* or "reporting guidance" or reporting standard* or reporting framework* or "reporting implementation" or "implementation reporting" or "reporting intervention" or "intervention reporting" or reporting checklist* or "reporting completeness") and (programme or program or programs or programmes or intervention or interventions or implementation or "implementation research" or study or studies) and (Reproductive health* or sexual health* or maternal health* or maternal or sexual or sexuality or reproductive or "public health" or health or medical or epidemiology)).af. |
