# Supplementary material for: A Systematic Review of Reporting Tools Applicable to Sexual and Reproductive Health Programmes: Step 1 in Developing Programme Reporting Standards
Source: PLoS One. 2015 Sep 29;10(9):e0138647. doi: 10.1371/journal.pone.0138647 (PMC4852887; doi:10.1371/journal.pone.0138647)
Supplement: S1 Text — (PDF) [file pone.0138647.s003.pdf]

PROGRAMME REPORTING STANDARDS (PRS) FOR SEXUAL AND REPRODUCTIVE HEALTH

July 2014 (last revised August 2015)

Study Protocol describing systematic review, consensus process and user-testing

Department of Reproductive Health and Research

World Health Organization, Geneva

Unpublished

TABLE OF CONTENTS

1. Introduction 2
2. Goal and Objectives 2
3. Project components 3
4. Part I: Systematic Review 4
   - 4.1 Objective of the systematic review 4
   - 4.2 Criteria for considering reporting tools/guidance for this review 4
     - 4.2.1 General inclusion/exclusion criteria 4
     - 4.2.2 Type of programmes and outcomes 4
     - 4.2.3 Type of participants 5
   - 4.3 Search strategy for identification of tools/guidance 5
     - 4.3.1 Electronic databases searching 6
     - 4.3.2 Other electronic searching 6
     - 4.3.3 Personal contacts ..... Error! Bookmark not defined.
   - 4.4 Methods of the Systematic Review 6
     - 4.4.1 Title and abstract review 6
     - 4.4.2 Data extraction 7
     - 4.4.3 Data management 7
     - 4.4.4 Pilot review 7
     - 4.4.5 Review of essential elements for programme reporting 7
   - 4.5 Description of tools/guidance 8
5. Part II: Drafting of PRS guidelines 8
6. PART III: WHO Technical Consultation 8
7. PART IV: Pilot testing of PRS guidelines 9
8. Timeline 9

Unpublished

1. INTRODUCTION

Reporting of the key implementation elements of programmes in sexual and reproductive health (SRH) is essential to understand the impact of the programmes, as well as to guide the efforts for future replication and scale-up. Indeed, readers of a programme publication need clear and complete information of the intervention process to be able to assess its evidence and quality as well as replicate the model. However, there is growing recognition that the key variables for understanding programme implementation – how, when, and under what conditions programmes are being implemented – is inadequately reported on in the scientific literature on SRH. Many programmes report on outcomes without describing their implementation process in greater detail. For example, in a recent systematic review conducted by the WHO and Johns Hopkins University on comprehensive adolescent health programs, it was found that the programme components, implementation and evaluation methodologies reported either in the peer reviewed literature or grey literature were inconsistent in depth and scope [1]. Consequently, programmes may demonstrate impact without providing any evidence on the process through which results were achieved. Lack of detail on the implementation process does not only limit the understanding of intervention vs. outcomes, but limits replication of the programme in other contexts.

In 1996, the lack of adequate reporting on randomized clinical trials spurred the development of the Consolidated Standards of Reporting Trials (CONSORT). Chalmers and Glasziou reported a significant amount of waste in reports of clinical research studies; over 30% of trial interventions were not sufficiently described and over 50% of planned outcomes were not reported in this analysis [2]. Since then, the CONSORT statement has been endorsed by over 400 journals in multiple languages and gone through several revisions (most recent in 2010) [3]. So too, other guidelines have been developed to assist design and reporting of health research, such as the STROBE (observational studies), PRISMA (systematic reviews and meta-analysis) and COREQ (qualitative research) guidelines. However, none of these guidelines were designed to report on the process through which programmes are being implemented [4]. Development of standardized guidelines for process documentation and programme reporting could therefore fill a significant gap in existing guidance and methods for implementation research.

To address this gap, the WHO is initiating a consultative process to review existing templates for process documentation and programme reporting, with the goal to develop Programme Reporting Standards (PRS) with a focus on SRH. Similar to the CONSORT guidelines, a checklist and flowchart format can be employed, describing key elements for understanding the implementation process. The intent is that the PRS will be used by programme staff and researchers to document and share how programmes were prepared, implemented and evaluated -- thus allowing others to replicate the model. It can also help facilitate the use of implementation research as part of programme implementation, since many of the processes and contextual variables documented through this process are useful sources of data for implementation research studies.

2. GOAL AND OBJECTIVES

The overall goal of the PRS project is to improve the quality of programme preparation, implementation and evaluation process reporting for programmes in SRH, by providing guidance on the essential elements to include when reporting on results from programmes (both in peer reviewed and grey literature). The PRS can help authors write programme reports and publications, as well as aid editors

Unpublished

and peer-reviewers in reviewing publications, and readers in critically appraising reports. It can also serve as guidance for SRH program preparation, implementation and evaluation.

The objectives of the PRS project are three-fold:

I. To provide an overview of available process reporting tools (used by researchers, organizations, donors) and reporting guidance (used by journals, similar to CONSORT) applicable to programmes in the field of SRH.
II. To develop PRS to be used by programme staff and researchers when reporting on programme preparation, implementation and evaluation processes in the field of SRH;
III. To assess the user-feasibility of the PRS for SRH programmes in a variety of platforms.

Note that the project *does not* seek to develop comprehensive guidelines for designing, developing and implementing SRH programmes. It merely seeks to provide guidance for *reporting* on programme processes and their results. However, clear guidance of what should be reported in publications can help program developers design and implement programs, too.

The PRS tool will be continuously updated as more evidence emerges, in line with recommendations from a core technical working group of experts in SRH programmes.

3. PROJECT COMPONENTS

The development of the PRS tool will follow guidelines for the development of reporting guidelines established by Moher et al. [5], and consist of the following steps:

I. A systematic review(s) on tools currently used for process documentation, program reporting and reporting guidance applicable to SRH programmes in order to identify core reporting items for potential inclusion in a PRS tool;
II. Further consolidate the list of items identified as part of the systematic review through a three-round Delphi consensus survey with experts in SRH program implementation, and experts in the development of reporting standards.
III. Draft a PRS tool based on the results from the Delphi exercise.
IV. Convene a WHO technical consultation with a core group of experts, including different stakeholders (i.e., researchers and programme implementers in the field of SRH, major public health journals, donors) to review the current evidence on programme reporting, implementation reporting tools and provide feedback on the draft PRS tool;
V. Assess the user-feasibility of the draft PRS tool among organizations implementing SRH programmes in different regions globally.
VI. Revise the PRS based on the feedback received from the user testing into a final version ready for launch.

The PRS project will be carried out between June 2014 and June 2016. It will be funded and implemented by the Department of Reproductive Health and Research, WHO and carried out in collaboration with the Alliance for Health Policy and Research at the WHO, and consultant Ms. Anna Kågesten at Johns Hopkins Bloomberg School of Public Health.

The purpose of this protocol is to guide the conduct of the main steps of the project (as outlined above) and will serve as a blueprint for the project methodology.

Unpublished

4. PART I: SYSTEMATIC REVIEW

4.1 OBJECTIVE OF THE SYSTEMATIC REVIEW

The first step of the project is to carry out a systematic review, including two major parts:

I. Provide an overview of current reporting guidance (similar to CONSORT) and other tools that have been used, or are suitable to use, for process documentation and programme reporting in the field of SRH;
II. Identify the core reporting items (focusing on the programme preparation, implementation and evaluation process) that need to be reported on for SRH programmes, and thus should be included in the PRS tool.

The process will follow a cascading approach, where core items will be extracted from each included tool. That is, we will first identify relevant reporting tools or guidance and subsequently review these for relevant items.

4.2 CRITERIA FOR CONSIDERING REPORTING TOOLS FOR THIS REVIEW

4.2.1 General inclusion/exclusion criteria

For the purpose of this review, we are interested in providing an overview of reporting guidelines, checklists, narrative guidance documents or other tools ("tools" for short) applicable to the reporting of programme preparation, implementation and evaluation processes in the field of SRH.

As part of this review, all identified reporting tools irrespective of language that were published between January 2000 and July 2014 will be assessed. The review process will consider the following general inclusion criteria for reporting tools during the title and abstract screening:

- All article/document types.
- Describe a tool or provide recommendations relevant to programme reporting (any mode/methods)
- Published in all parts of the world.
- Published in peer-reviewed or grey literature.
- Published between January 2000 and July 2014.

Tools/guidance will be excluded if:

- There is no reference source;
- It has never been used in practice;
- The tools/guidance is a minor modification of an already established tool.

4.2.2 Type of programmes and outcomes

Reporting tools have to be applicable/relevant to programmes in the field of SRH. This does not necessarily mean that the tools should have been developed specifically for these areas, but the tools should be possible to apply to the reporting of SRH programmes. Examples of outcome areas that can be addressed by such programmes include:

Unpublished

- Maternal mortality and morbidity
- Abortion
- Sexually transmitted infections
- HIV/AIDS prevention and treatment
- Adolescent pregnancy
- Family planning/contraception
- Sexual well-being
- Gender inequalities and gender norms
- Sexual abuse, harassment and violence, including intimate partner violence (IPV)
- Lesbian, bi, gay, transsexual and queer issues (LGBTQ)

We choose not to limit the search by programme or study design since many different designs can contain the information that we are searching for. The review steps will be inclusive so as to capture as many relevant tools as possible.

4.2.3 Type of participants

The PRS will focus on programmes focused on adolescent (10-19), youth (15-24) or adult (18+) participants. Programmes targeting young children (including new-borns) are beyond the scope of the current review.

4.3 SEARCH STRATEGY FOR IDENTIFICATION OF TOOLS/GUIDANCE

Reviewers will be able to read titles and abstracts English, French, Spanish, Portuguese, Turkish, Swedish, Norwegian and Danish. Records in other languages will be translated into English.

We will develop a core strategy for PubMed using specific MeSH terms and text words defined a priori. This strategy will form the basis for the strategies developed for other electronic search processes (see Table 1).

Table 1. Draft search strategy for PubMed

**1) Reporting tool/guidance**	
Key words:	Reporting guideline*[tw] OR reporting tool*[tw] OR reporting guidance[tw] OR reporting standard*[tw] OR reporting framework*[tw] Reporting of implementation*[tw] OR reporting of intervention*[tw] OR reporting checklist*[tw] OR completeness or reporting[tw]
Controlled vocabulary:	Checklist*[Mesh] OR Publishing/standards*[Mesh] OR
**AND**	
**2) Programme/intervention**	
Key words:	Programme[tw] OR program[tw] OR programmes[tw] OR programs[tw] OR intervention[tw] OR interventions[tw] OR implementation[tw] OR implementation research[tw] OR study[tw] OR studies[tw]

Unpublished

Controlled vocabulary:	Program development*[Mesh]
**AND**	
**3) Applicable to maternal, sexual and reproductive health**	
Key words:	Reproductive health*[tw] OR sexual health*[tw] OR sexuality[tw] OR sexual[tw] OR reproductive[tw] OR maternal[tw] OR maternal health*[tw] OR public health[tw] OR health[tw] OR medical[tw] OR epidemiology[tw]
Controlled vocabulary:	Reproductive Health*[Mesh]

4.3.1 Electronic databases searching

The following electronic databases will be searched from year 2000 to July 2014:

- PubMed
- Scopus
- PsycInfo
- EMBASE/MEDLINE
- Global Health

4.3.2 Other electronic searching

A grey literature search will be conducted, which due to logistical constraints will be focused on donors to HRP. These include:

- USAID
- MERCK
- The Bill and Melinda Gates Foundation
- The MacArthur Foundation
- The David and Lucile Packard Foundation

We will also search the EQUATOR Network library for health reporting, focused on implementation process reporting tools.

4.4 METHODS OF THE SYSTEMATIC REVIEW

4.4.1 Title and abstract review

The study team will screen all titles retrieved in the search process. All titles thought to address the study objective will be promoted to the abstract review phase. Abstracts will be reviewed using the screening questions provided in Table 2. In the absence of abstracts, table of contents and summaries in reports will be used. Articles that pass abstract review will be promoted to full text review.

Table 2: Abstract Screening Questions

**SCREENING QUESTIONS**	**NO**	**YES**

Unpublished

1. Is the article published in a peer-reviewed journal and/or grey literature source? Does the article describe a programme reporting tool or guidance?<br>2. Can the tool/guidance be applied to programmes that target SRH?<br>3. Has the tool/guidance been published after year 2000?<br><br>*NOTE: If any answer falls into a "No" for Question 2-4 the article will be excluded.*		
**Include for full-text review**		

4.4.2 Data extraction

All records identified by the electronic search strategies will be evaluated initially according to the screening form (see Table 2) on the basis of the titles and abstracts. Irrelevant records will be discarded. Full text of studies of relevant articles will then be obtained, as will articles whose available citations did not provide sufficient information to decide.

After obtaining full-text for included articles, data will be extracted using the form listed in Appendix I. Articles excluded at this stage will be listed separately with the reason for exclusion stated. The same reviewers will extract data; when inconsistencies are identified or disagreements occur they will be resolved through discussion or consulting an independent reviewer. Attempts will be made to contact authors to obtain missing information or clarification whenever necessary.

4.4.3 Data management

The review will use Endnote bibliographic software to store and keep track of citations. A shared library will be created between the reviewers. Electronic searches will be downloaded directly into Endnote while studies retrieved from other sources will be entered in Endnote manually. Duplicates will be deleted and each study will be assigned a unique identification number for the review.

4.4.4 Pilot review

Before initiating the formal review, the core search strategy, screening questions and data-extraction form will be piloted during August 2014. The search strategy will encompass the entire time period (2000-2014) and two reviewers will scan the identified articles according to the screening form. Two independent reviewers will scan a random sample of these (total of 50 references) in duplicate, in order to test inter-observer variability. When inconsistencies are identified or disagreements occur they will be resolved through discussion or consulting a third reviewer.

The same reviewers will then extract data from all identified studies. When inconsistencies are identified or disagreements occur they will be resolved through discussion or consulting a third reviewer. This will enable identification of flaws or gaps in the data-extraction form. A final version of the form, including any modifications will be prepared after piloting.

The search strategy listed in Table 1 will be adopted according to database standards and used in the pilot review.

4.4.5 Review of core items for programme reporting

Unpublished

The second objective of the systematic review is to identify core items for potential inclusion in a PRS tool. This will be done as a parallel process to the identification of reporting tools. Items reported on will be summarized for each included reporting tool. During this process, additional input will be sought from relevant articles on evaluation or implementation research in general. These elements will be complied into a final list of suggested items to report on.

4.5 DESCRIPTION OF TOOLS

A table with the characteristics of the included tools/guidance will be prepared. This table will show the tools/guidance by type of organization (implementer, donor, journal) and year of use. The table will also list the main area for programme reporting, and the key elements reported on.

5. PART II: DELPHI CONSENSUS SURVEY

Following the systematic review, the list of identified items will form the basis for a Delphi consensus survey. Originally developed by the Rand Corporation, the Delphi survey technique (Delphi for short) is a method to explore, seek consensus and correlate judgments on a specific topic [6]. Specifically, the opinion of experts is sought through an iterative series of structured survey rounds where each expert participant completes an anonymous questionnaire. Throughout the process, the responses and feedback from the participants is fed into the next round until consensus has been reached. That is, the results from each round is complied and communicated back to participants as part of the next round. Participants thus become aware of the collective opinion of the group, and also have the opportunity to change their opinion. The Delphi has been commonly applied in medical and health services and many versions of this technique currently exist. We will use a modified Delphi process guideline developed by Hasson et al. [6], and also build on recommendations made by the CONSORT team around conducting Delphi exercises [7]. The Delphi is tentatively planned to start in August, with the last round administered and analyzed in November 2015.

A panel of 50-100 experts from implementing governmental and non-governmental organizations, donors, bilateral and multilateral organs, and experts in the development of reporting standards will be invited to participate. Participants will be asked to respond to an iterative series of three survey rounds. During each round, they will be asked about the relevance of items for potential inclusion in a PRS tool. During the first round, participants will be able to suggest additional items and modification to the structure and language of items and their descriptions. Participants will have three weeks to complete the first round of the survey, and two weeks to complete rounds two and three respectively. There will be approximately two weeks in between each round.

Participants will be instructed that their participation is entirely voluntary and that they will be able to withdraw from the process at any time. All data collected will be kept completely confidential and results will be anonymous when reported back to the group.

6. PART III: WHO TECHNICAL CONSULTATION

The objective of the technical consultation is to get expert feedback on the draft PRS guidelines in order to develop a final tool ready for piloting.

Unpublished

The consultation will include technical experts in programme reporting, implementation research and SRH programmes. In total, the consultation is expected to last no more than 2 days. In preparation for the meeting, participants will receive a background document including the systematic review, the draft PRS tool, and results from the Delphi exercise.

7. PART IV: PILOT TESTING OF PRS GUIDELINES

The final step of the project is to assess the usability of the draft PRS tool for programme reporting in the field of SRH.

User-testing and piloting will be done through various programmes supported by HRP partners. Programmes selected will have equal representation from different geographical regions, and different sub-fields within SRH. Programmes will be selected with input from the technical working group, after which contact will be made with the program director or officer. Piloting should ideally be done with new programmes for which publications and reports are yet to be written. An additional protocol describing the full piloting process in greater detail will be created following the technical consultation.

8. TIMELINE

	**2014**							**2015**				**2016**		
**Task**	**June**	**July**	**Aug**	**Sept**	**Oct**	**Nov**	**Dec**	**Jan-Mar**	**Apr-June**	**July-Sept**	**Oct-Dec**	**Jan-Mar**	**Apr-June**	**July**
Create study protocol	XXX													
Set search criteria	X													
Pilot review		XX												
Finalize search criteria		XX	XXX											
Search			X	XX										
Peer reviewed title and abstract screen				XXX										
Finalize list of peer reviewed articles				X	XX									
Full text data extraction peer reviewed articles					XXX									
Grey literature title and abstract screen					XXX									
Data extraction grey literature						XXX								
Reach out to authors and experts as needed				XXX	XXX	XXX								
Finalized list of tools/guidance to include						XXX								
Identify reporting items				XXX	XXX	XXX								
Draft and submit manuscript							XXX	XXX	XXX					
Delphi survey										XXX	XX			
Technical consultation												XXX		
Revise tool												XX	XXX	
Assess usability, finalize tool													XXX	XXX

Unpublished

APPENDIX I: DATA EXTRACTION FORM

#	Question	Response codes
	If from the grey literature, please list the URL where the publication was found	Write answer
1	Article identification number	# |___|___|___|___|
2	Data abstractor name	Write answer
	**STUDY IDENTIFIERS AND BACKGROUND**	
3	Date of extraction (dd/mm/yy)	|___|___|___|___|___|___| d d m m y y
4	Author/organization	
5	Article Title	Write answer
6	Tool/guidance name (if applicable)	Write answer
7	Type of Publication Source	(1) Grey<br>(2) Peer reviewed<br>(3) Personal communication
8	Website of publication (if applicable)	
9	Year of publication	Write answer
10	Journal Name (if applicable)	Write answer
11	Country/ies	Write answer
12	Publication language	(1) English<br>(2) French<br>(3) German<br>(4) Chinese<br>(5) Spanish<br>(6) Russian<br>(7) Portuguese<br>(8) Other
	**TOOL CHARACTERISTICS**	
14	Purpose of tool (what was the tool developed for); i.e. methodologies, subject area	Write answer
15	Programme subject area targeted (if applicable)	1) Maternal mortality and morbidity<br>2) Abortion<br>3) Sexually transmitted infections<br>4) HIV/AIDS prevention and treatment<br>5) Adolescent pregnancy<br>6) Family planning/contraception<br>7) Sexual well-being<br>8) Gender inequalities and gender norms

Unpublished

		9) Sexual abuse, harassment and violence, including intimate partner violence (IPV)<br>10) Lesbian, bi, gay, transsexual and queer issues (LGBTQ)<br>11) Other ________________________<br>12) Not applicable
16	Nr of items included in checklist (as applicable) and their description	Write answer
17	Nr of items specific to implementation (as applicable)	Write answer
19	Other important details	Write answer

Unpublished

References

1. Kågesten A, Tunçalp Ö, Ali M, Chandra-Mouli V, Tran N, et al. (2015) A Systematic Review of Reporting Tools applicable to Sexual and Reproductive Health Programmes: Step 1 in Developing Programme Reporting Standards. Forthcoming in PLOS One.
2. Chalmers I, Glasziou P (2009) Avoidable waste in the production and reporting of research evidence. Lancet 374: 86-89.
3. Moher D, Hopewell S, Schulz KF, Montori V, Gotzsche PC, et al. (2010) CONSORT 2010 Explanation and Elaboration: Updated guidelines for reporting parallel group randomised trials. J Clin Epidemiol 63: e1-37.
4. Peters D, Tran N, Adam T (2013) Implementation Research in Health: A practical guide. Alliance for Health Policy and Systems Research. World Health Organization.
5. Moher D, Schulz KF, Simera I, Altman DG (2010) Guidance for developers of health research reporting guidelines. PLoS Med 7: e1000217.
6. Hasson F, Keeney S, McKenna H (2000) Research guidelines for the Delphi survey technique. Journal of advanced nursing 32: 1008-1015.
7. Hopewell S, Clarke M, Moher D, Wager E, Middleton P, et al. (2008) CONSORT for reporting randomized controlled trials in journal and conference abstracts: explanation and elaboration. PLoS Med 5: e20.
